# Caveolae, Fenestrae and Transendothelial Channels Retain PV1 on the Surface of Endothelial Cells

**DOI:** 10.1371/journal.pone.0032655

**Published:** 2012-03-05

**Authors:** Eugene Tkachenko, Dan Tse, Olga Sideleva, Sophie J. Deharvengt, Marcus R. Luciano, Yan Xu, Caitlin L. McGarry, John Chidlow, Paul F. Pilch, William C. Sessa, Derek K. Toomre, Radu V. Stan

**Affiliations:** 1 Department of Pathology, Dartmouth Medical School, Lebanon, New Hampshire, United States of America; 2 Department of Microbiology and Immunology, Dartmouth Medical School, Lebanon, New Hampshire, United States of America; 3 Heart and Vascular Research Center, Dartmouth Medical School, Lebanon, New Hampshire, United States of America; 4 Norris Cotton Cancer Center, Dartmouth Medical School, Lebanon, New Hampshire, United States of America; 5 Department of Pharmacology, Yale University, New Haven, Connecticut, United States of America; 6 Department of Cell Biology, Yale University, New Haven, Connecticut, United States of America; 7 Department of Biochemistry, Boston University, Boston, Massachusetts, United States of America; 8 Department of Medicine, University of California, San Diego, California, United States of America; Institut Curie, France

## Abstract

PV1 protein is an essential component of stomatal and fenestral diaphragms, which are formed at the plasma membrane of endothelial cells (ECs), on structures such as caveolae, fenestrae and transendothelial channels. Knockout of PV1 in mice results in *in utero* and perinatal mortality. To be able to interpret the complex PV1 knockout phenotype, it is critical to determine whether the formation of diaphragms is the only cellular role of PV1. We addressed this question by measuring the effect of complete and partial removal of structures capable of forming diaphragms on PV1 protein level. Removal of caveolae in mice by knocking out caveolin-1 or cavin-1 resulted in a dramatic reduction of PV1 protein level in lungs but not kidneys. The magnitude of PV1 reduction correlated with the abundance of structures capable of forming diaphragms in the microvasculature of these organs. The absence of caveolae in the lung ECs did not affect the transcription or translation of PV1, but it caused a sharp increase in PV1 protein internalization rate via a clathrin- and dynamin-independent pathway followed by degradation in lysosomes. Thus, PV1 is retained on the cell surface of ECs by structures capable of forming diaphragms, but undergoes rapid internalization and degradation in the absence of these structures, suggesting that formation of diaphragms is the only role of PV1.

## Introduction

Caveolae, fenestrae and transendothelial channels (TEC) are endothelial structures involved in microvascular permeability [1], [2], [3], [4], [5]. In the ECs of capillaries of visceral organs, these structures are provided with diaphragms [1], [6], [7]. The only known structural component of the diaphragms is PV1 [8], [9], [10], [11], [12], a vertebrate protein encoded by the *Plvap* gene [1], [11], [13]. Knockdown of PV1 in ECs in culture results in the disappearance of all diaphragms [10], [11], [12]. Knockout of PV1 in mice also causes the disappearance of all diaphragms and results in *in utero* and perinatal mortality due to impairment of vascular permeability [14].

Our understanding of the complex phenotype occurring in PV1−/− mice would be strengthened by the knowledge of whether the diaphragm formation is the only cellular role played by PV1. We addressed this question by measuring the effect of removal of endothelial structures capable of forming diaphragms on the cellular PV1 protein level. PV1 and the diaphragms are present only in ECs of microvessels (*i.e*. capillaries and venules) of visceral organs. For the *in situ* approach, our analysis was focused on microvessels in two types of vascular beds such as the lung and the kidney. Lung capillaries are of a continuous type and their ECs have only caveolae but no fenestrae or TEC [1]. Conversely, kidney capillaries are of a fenestrated type, their ECs being provided with fenestrae and TEC in great excess to caveolae [1], [15].

We showed that deletion of caveolae by knockout of their components Cav1 [16], [17], [18] or PTRF/cavin-1 [19], [20] resulted in the dramatic decrease of PV1 protein level in lung microvascular ECs, which lacked any structures capable of forming diaphragms. We determined that the reduction in PV1 protein level was due to increased internalization rate via a clathrin- and dynamin-independent pathway followed by degradation in lysosomes. In contrast to lungs, the absence of caveolae caused only slight reduction in PV1 protein level in fenestrae- and TECs-rich microvascular ECs of kidneys. Therefore, PV1 is retained on the surface of microvascular ECs by structures capable of forming diaphragms. In the absence of these structures, PV1 undergoes rapid internalization and degradation suggesting that formation of diaphragms is the only function of PV1 protein.

## Results

### Protein level of PV1 is maintained by the presence of structures capable of forming diaphragms *in vivo*

**Figure 1:**
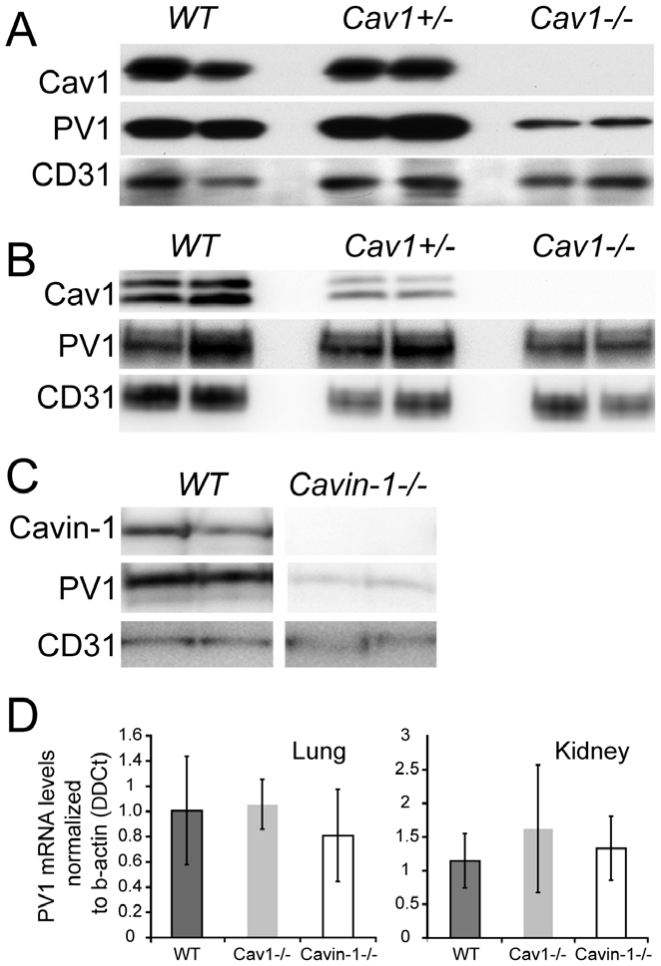
Protein level of PV1 is maintained by the presence of caveolae *in vivo*. A–B) Protein levels of PV1, Cav1 and CD31 in the lung (A) and kidney (B) total membranes of Cav1−/−, Cav1+/− and WT mice were detected by immunoblotting. C) Protein levels of PV1, cavin-1 and CD31 in the lung total membranes of cavin-1−/− and WT mice were detected by immunoblotting. D) PV1 mRNA levels in the lung (*left panel*) and kidney (*right panel*) of WT, Cav1−/− and cavin-1−/− mice.

Knockout of Cav1 in mice (Cav1−/−) led to the decrease (84.4%+/−7%) in PV1 protein level in the lungs (Fig. 1A), in accord with a previous report [21]. This decrease was not due to a lower number of ECs in Cav1−/−, as indicated by similar protein levels of endothelial marker CD31/PECAM-1 [22], [23] in Cav1−/−, Cav+/− and WT (Fig. 1A). A similar reduction (76.6+/−11%) in PV1 protein level in the lungs was observed for knockouts of cavin-1 (cavin-1−/−) (Fig. 1B). In contrast to lungs, the removal of caveolae in kidneys by knockout of Cav1 caused only a slight reduction (17.5%+/−3) in protein level of PV1 (Fig. 1C). PV1 mRNA levels in lungs and kidneys were similar among WT, Cav1−/− and cavin-1−/− mice (Fig. 1D), indicating that transcription of PV1 is not affected by the lack of caveolae.

**Figure 2:**
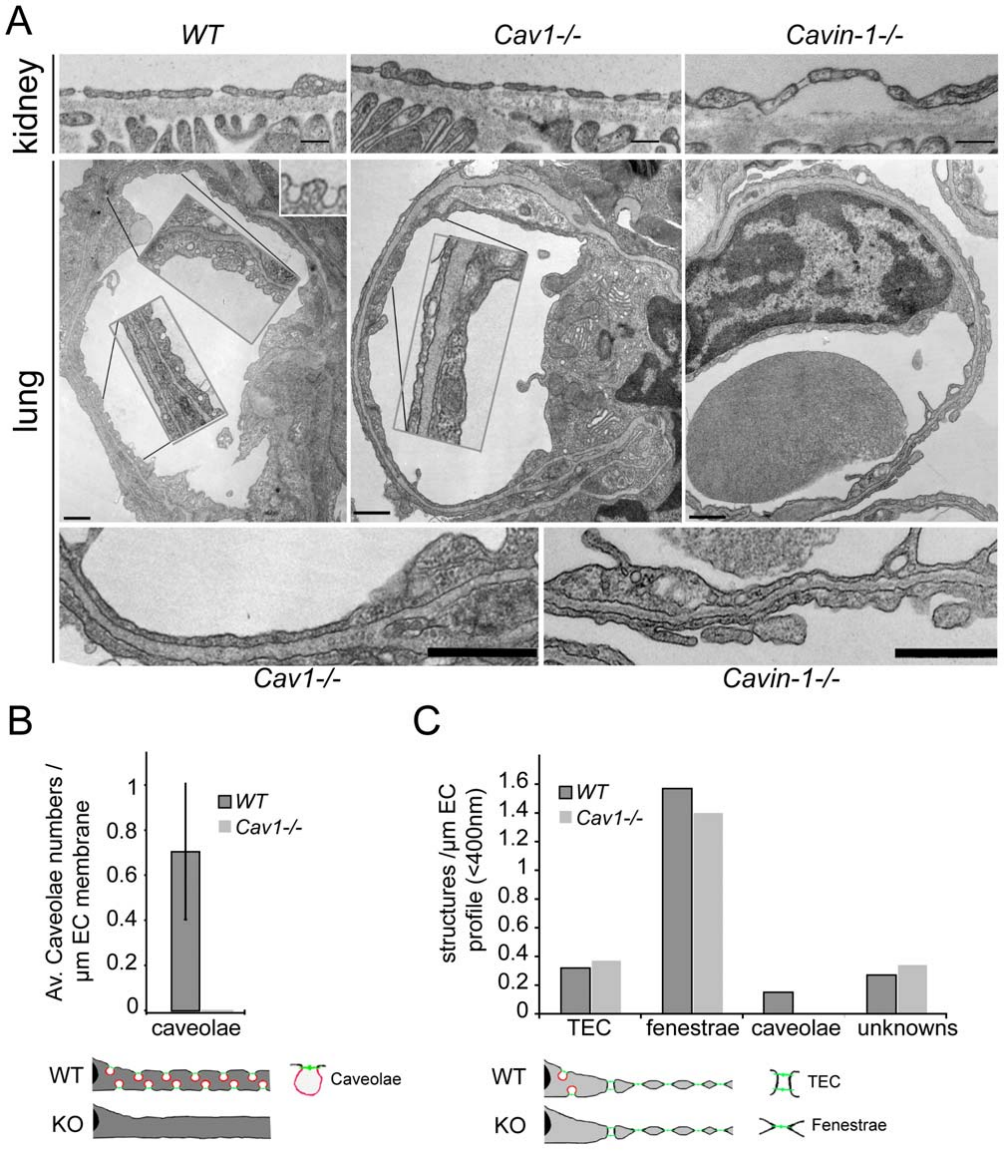
Protein level of PV1 correlates with the number of structures capable of forming diaphragms *in vivo*. A) Electron micrographs of capillary ECs of the kidneys (*top panels*) and lungs (*middle and bottom panels*) of WT, Cav1−/− and cavin-1−/− mice, as indicated. TEC and fenestrae are present in the kidneys of WT, Cav1−/− and cavin-1−/− mice (*top panels*). (*Middle and bottom panels*) Caveolae with stomatal diaphragms are present in the lungs of WT and absent in Cav1−/− and cavin-1−/− mice (*middle panel*). Insets in *middle panels* are a 2-fold magnification of the noted stretches of ECs. Bottom panels are a 3-fold magnification of ECs of Cav1−/− (*left*) and cavin-1−/− (*right*). Bars −200 nm. B) Morphometric analysis of the number of lung endothelial caveolae in WT and Cav1−/− mice demonstrating the absence of caveolae in the latter. C) Morphometric analysis of the numbers of kidney endothelial TEC, fenestrae and caveolae in WT and Cav1−/− mice.

We employed transmission electron microscopy (TEM) to get a better understanding of structural differences between the microvasculature of lungs and kidneys in the context of diaphragm formation. In concordance with previous studies [8], [9], [24], [25] (reviewed in [1]), ECs in lungs of WT formed caveolae provided with diaphragms but did not form fenestrae or TEC (Fig. 2A, *left lower panels*). The removal of caveolae by knockout of either Cav1 or cavin-1 resulted in lung microvasculature completely devoid of all structures capable of forming diaphragms (Fig. 2A, *middle and right lower panels*). In contrast to the lungs, ECs in the peritubullar capillaries of kidneys contained many fenestrae and TEC provided with diaphragms [15] (Fig. 2A, *left upper panel*). Removal of caveolae did not affect the ability of ECs in kidney peritubullar capillaries to form these structures (Fig. 2A, *middle and right upper panels*). Morphometric analysis confirmed on one hand the absence of caveolae in the microvasculature of Cav1−/− lungs (Fig. 2B) and kidneys (Fig. 2C), and on the other hand it showed that the densities of fenestrae and TEC in the kidneys of Cav1−/− and WT mice were similar (Fig. 2C). Thus, removal of caveolae caused a complete absence of structures capable of forming diaphragms and low PV1 protein level in the microvasculature of the lungs. In contrast, the microvasculature of the kidneys of Cav1−/− mice had high numbers of structures capable of forming diaphragms (fenestrae and TEC) and high PV1 protein level.

### Protein level of PV1 is maintained by the presence of caveolae *in vitro*

**Figure 3:**
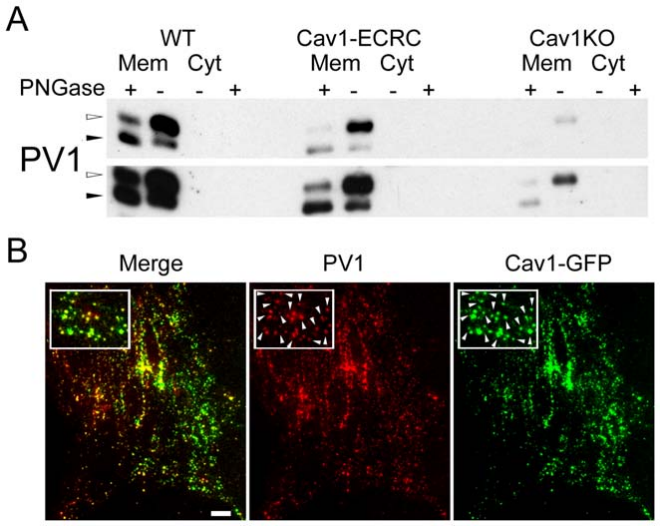
Protein level of PV1 is maintained by the presence of caveolae *in vitro*. A) Protein levels of PV1 in MLEC-wt(*WT*), MLEC-Cav1KO (*Cav1KO*) and MLEC-Cav1-ECRC (*ECRC*) cells detected by immunoblotting with anti-PV1 antibodies. *M* - Corresponds to membrane proteins, *C* – cytosolic proteins. Equal amount of membrane protein was loaded whereas the cytosolic proteins were normalized to membrane extract volume. The membrane and cytosolic proteins were also partially deglycosylated with PNGase F (*+*), which resulted in the drop in PV1 molecular weight. Note very low PV1 level in Cav1KO cells and increased PV1 protein level in cells reconstituted with Cav1 (Cav1-ECRC). The top and bottom panels are different exposures of the same blot. B) PV1 is predominantly associated with caveolae on the surface of lung endothelial cells. PV1 (*red*) colocalizes with Cav1-EGFP (*green*) at the plasma membrane of live MLEC, as shown by TIRFM. Insets demonstrate the extensive colocalization of the two labels (*white arrowheads*). Scale bars −20 µm.

To investigate the mechanism by which structures capable of forming diaphragms maintain PV1 protein level, we employed mouse lung endothelial cells (MLECs) which, when isolated from WT (WT) mice, form caveolae but not fenestrae or TEC [26], [27]. Caveolae do not form in MLECs isolated from Cav1−/− mice (Cav1KO), but form in MLECs isolated from Cav1−/− mice in which Cav1 expression was reconstituted with transgenic expression of canine Cav1 under the control of the endothelial-specific preproendothelin promoter (Cav1ECRC) [26], [27]. The amount of PV1 protein translated and matured to fully N-glycosylated form in MLEC-Cav1-ECRC was similar to that in the MLEC-WT and higher than in MLEC-Cav1KO (Fig. 3A). Therefore, the PV1 protein level in MLEC *in vitro* is maintained by the presence of caveolae.

The direct correlation between the presence of caveolae and PV1 protein level in lung ECs suggests that PV1 predominantly associates with caveolae on the cell surface of MLEC-WT. We examined the specificity of PV1 association with caveolae in MLEC-WT using two-color total internal reflection fluorescent microscopy (TIRFM). TIRFM allowed us to visualize the fluorescent signal only in the coverglass-proximal region of the cell (≈100 nm calculated penetration angle) mostly corresponding to plasma membrane. Live MLEC-WT expressing Cav1-GFP were labeled at 4°C with fluorophore tagged anti-PV1 antibodies and imaged at 37°C immediately thereafter. PV1 predominantly associated with caveolae as indicated by extensive co-localization of PV1 with Cav1-GFP (*white arrows*, Fig. 3B).

### Absence of caveolae does not affect the transcription and translation rates of PV1

**Figure 4:**
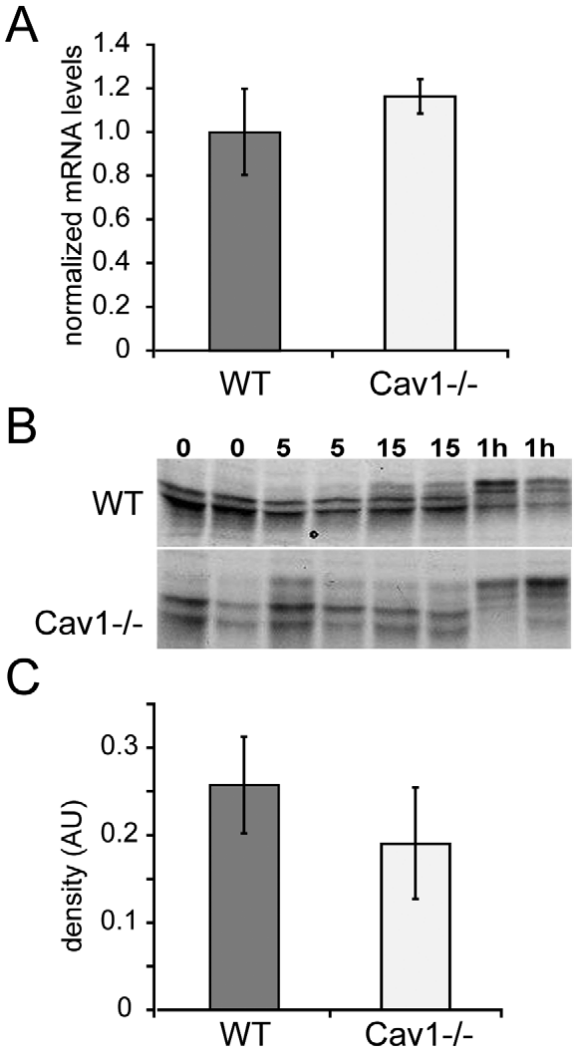
Absence of caveolae in lung ECs does not affect transcription and translation levels of PV1. A) PV1 mRNA levels in MLEC-wt (*WT*) and MLEC-Cav1KO (*Cav1KO*) cells measured by real time quantitative PCR. The data was obtained from quadruplicate samples and normalized to b-actin mRNA levels (ΔΔCt). Bars – SEM. B) Pulse ^35^S metabolic labeling of MLEC-WT (*top panel*) and MLEC-Cav1KO (*bottom panel*) cells followed by PV1 immunoprecipitation at the indicated time points and ^35^S fluorography. Duplicate samples are shown for each time point assessed. PV1 has four active N-glycosylation sites and therefore shows five bands, the lowest representing the non-glycosylated form and the four higher bands representing various degrees of N-glycosylation. C) Densitometric quantitation of the amount of PV1 translated after 10 min pulse with ^35^S-methionine and cysteine in MLEC-WT and MLEC-Cav1KO cells. Error bars correspond to SEM (n = 3).

Reduction in total PV1 protein levels in ECs lacking caveolae could be explained by defects in transcription or translation of PV1. We examined this possibility by determining the effect of Cav1 knockout on PV1 mRNA level and translation rate of PV1 protein in lung ECs isolated from wild type (MLEC-WT) and Cav1−/− (MLEC-Cav1KO) mice. The level of PV1 mRNA normalized to beta-actin mRNA was similar in MLEC-WT and MLEC-Cav1KO (Fig. 4A), in agreement with *in vivo* data (Fig. 1D). Thus, deletion of Cav1 does not affect PV1 mRNA level in ECs.

The translation rates of PV1 mRNA into protein were measured by pulse metabolic labeling of MLEC-WT and MLEC-Cav1KO cells with ^35^S-methionine and ^35^S-cysteine. Immunoprecipitated and SDS-PAGE resolved ^35^S-labeled PV1 appeared as five bands by fluorography, representing the non-glycosylated, N-glycosylation intermediates and fully N-glycosylated forms of PV1 polypeptide. PV1 has four functional N-glycosylation sites [8] that were confirmed by point mutagenesis (D. Tse, R. Stan, manuscript in preparation). The amount of PV1 protein translated and matured to fully N-glycosylated form in the MLEC-Cav1KO was similar to the WT cells (Fig. 4B–C), demonstrating that Cav1 absence has no effect on the translation rate of PV1 in lung ECs.

### PV1 is retained on the surface of lung endothelial cells by caveolae

**Figure 5:**
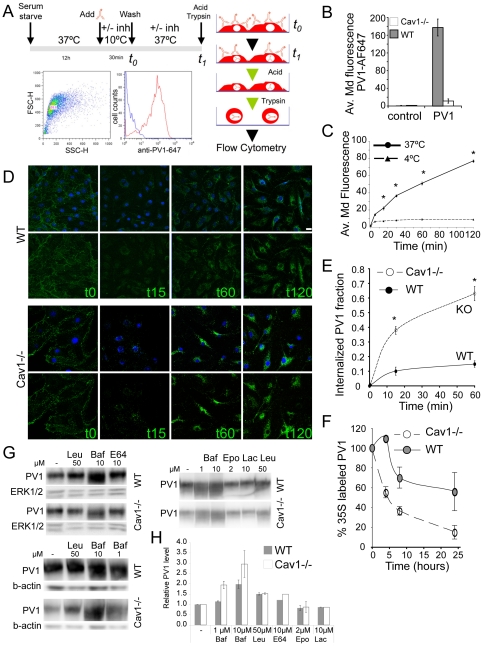
PV1 is retained on the surface of lung endothelial cells by caveolae. A) Schematic of the timeline (*upper right*) and the principal steps of PV1 internalization flow cytometric assay (*right*). An example of data gating and fluorescence intensity histogram is given in the lower left panels. B) Amount of PV1 on the surface of MLEC-wt (*WT*) and MLEC-Cav1KO (*Cav1 KO)* at *t_0_* expressed as median fluorescence intensity per cell from fluorophore-labeled anti-PV1 (*PV1*). Labeling of cells with isotype control non-immune antibodies showed the level of unspecific binding (*control*) (error bars correspond to stdev, n = 4, *p<0.01). C) Amount of internalized PV1 at different time points in MLEC-WT at 37°C (*solid line*) and 4°C (*dashed line*) expressed as median fluorescence intensity per cell from fluorophore-labeled anti-PV1 (*PV1*) (stdev, n = 6, *p<0.01). D) PV1 internalization in MLEC-WT (*WT, top panels*) and MLEC-Cav1KO (*Cav1 KO, bottom panels)* cells at different time points, as detected by confocal microscopy. Images are maximum projections of confocal stacks in green channel (PV1, *lower panels*) or green merged with blue (nuclei labeled with DAPI, *upper panels*). E) Internalization rate of PV1 in MLEC-WT (*solid line, solid circles*) and Cav1KO (*dashed line, open circles*) cells, expressed as a percentage from the total amount of PV1 on the cell surface. (stdev, n = 4 per time point, *p<0.01). F) Degradation curves of ^35^S labeled PV1 in MLEC-Cav1KO (Cav1KO, *dashed line*, *open circles*) and MLEC-WT (WT, *solid circles*), isolated from Cav1−/− and wild type mice, respectively. Data is representative of three experiments carried out in duplicate. G, H) PV1 degradation rates were measured in MLEC-WT (WT) and MLEC-Cav1KO (Cav1KO) treated with lysosomal or proteasomal inhibitors. G) Western blots used for densitometric quantifications of PV1 protein level. H) Quantitation of protein levels of PV1.

We hypothesized that the low PV1 protein level in lung ECs lacking caveolae may be explained by PV1 rapid internalization and degradation due to the absence of structures that can form diaphragms and retain PV1 on cell surface. We examined internalization rates of PV1 from the surface of MLEC-Cav1KO and MLEC-WT by flow cytometry (Fig. 5A). In accord with our hypothesis, the amount of PV1 on the surface of MLEC-Cav1KO was much lower than in MLEC-WT (Fig. 5B). PV1 was internalized in time-dependent manner (Fig. 5C) but the rate of PV1 internalization was significantly higher in MLEC-Cav1KO than in MLEC-WT (Fig. 5D–E). Therefore, absence of Cav1, a critical structural component of caveolae, resulted in an accelerated rate of PV1 internalization from the surface of lung ECs.

Next, we examined degradation rates of PV1 in MLEC-Cav1KO and MLEC-WT by using ^35^S metabolic labeling. The cells were pulsed with ^35^S-methionine and ^35^S-cysteine and a chase for 1, 4, 8 and 24 h allowed us to determine the half-life (t_1/2_) of ^35^S-labeled PV1 protein. In MLEC-Cav1KO cells, total cellular PV1 *t_1/2_* was in the range of ∼4 h, significantly shorter than in MLEC-WT (Fig. 5F). Therefore, the absence of caveolae in lung microvascular ECs resulted in a higher rate of degradation of the fully glycosylated PV1 protein.

To determine the mechanism of PV1 degradation we treated MLEC-WT and MLEC-Cav1KO with pharmacological inhibitors of either lysosomal or proteasomal degradation. Lysosomal enzymes were inhibited by treatment with 1 or 10 µM bafilomycin A1 (a V-ATPase inhibitor and inhibitor of lysosomal acidification [28]), 50 µM leupeptin (a serine and cysteine protease inhibitor) [29] or 10 µM E-64D (a membrane permeable cysteine protease inhibitor) [30] for 24 h and determination of PV1 levels by western blotting. Each of these inhibitors increased total cellular PV1 protein levels at 24 h (Fig. 5G). Inhibition of PV1 degradation had greater effect on protein level of PV1 in MLEC-Cav1KO than in MLEC-WT in agreement with an increased degradation rate of PV1 in the former cells. Inhibitors of the proteasomal pathway such as 2 µM epoxomycin [31] and 10 µM *clasto*-lactacystin β-lactone [32] had no effect on PV1 levels after 4, 8 or 24 h treatment (Fig. 5G–H and data not shown). Therefore, PV1 in MLECs is degraded in lysosomes.

### PV1 is internalized in clathrin- and dynamin- independent manner

**Figure 6:**
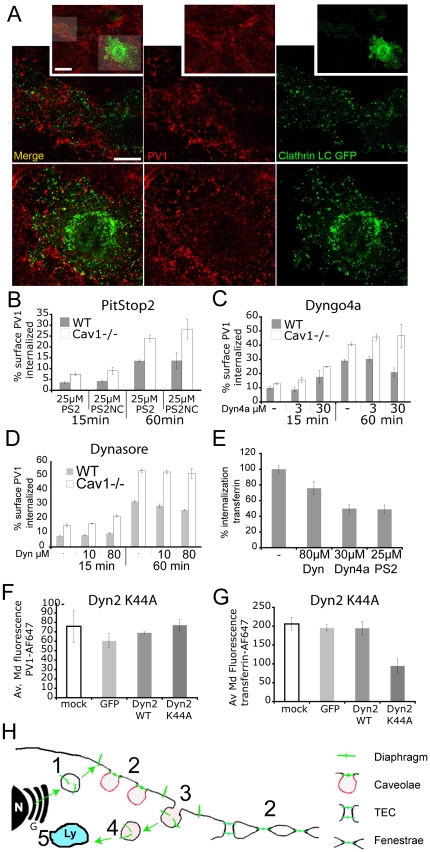
PV1 is internalized in clathrin and dynamin independent manner in WT and Cav1−/− cells. A) PV1 does not colocalize with clathrin-GFP on the cell surface. Confocal micrographs of MLEC-WT transfected with clathrin-GFP (*Clathrin, green*) and labeled with fluorescent anti-PV1 antibodies (*PV1, red*). The insets represent a low power field with two transfected cells. The areas in shaded in grey are magnified in lowed panels. B–G) PV1 and transferrin internalization rates in MLECs were quantified by flow cytometry. Error bars correspond to StDev. B–D) Percentage of fluorescent antibody labeled PV1 internalized from the cell surface. B) PV1 internalization at 15 and 60 min in presence and absence of the clathrin pathway inhibitor PitStop2 (*PS2*) or the inactive PitStop2 negative control (*NC*) (n = 4, *p*>0.05). C,D) PV1 internalization at 15 and 60 min in presence of dynamin inhibitors Dyngo-4a (C) (n = 4, *p*>0.05) or Dynasore (*D*) (n = 4, p>0.05). E) Median fluorescent intensity of transferrin-Alexa647 internalized within 10 min in the presence and absence of PitStop2, Dynasore or Dyngo4a (n = 4, *p<0.01). D–G) Internalization of PV1 (F) and transferrin (G) at 15 min in untransfected MLECs (mock) and MLECs transfected with eGFP-encoding plasmid (GFP), dynamin 2-eGFP fusion (Dyn2 wt) or dominant-negative form of dynamin 2 fused to eGFP (Dyn2 K44A MLECs (n = 4, *p<0.01). H) Schematic of PV1 (*green*) trafficking in ECs. *De novo* formed PV1 enters the secretory pathway and arrives at the cell surface by exocytosis (*green arrow*) using secretory vesicles (Step 1). On the plasma membrane PV1 is targeted to caveolae, fenestrae or TEC (Step 2) where it forms diaphragms. PV1 is internalized via clathrin- and dynamin-independent endocytic mechanism (*Step 3 and 4*) followed by degradation in the lysosomes (*Step 5*).

We examined the mechanism of PV1 internalization by studying its dependence on clathrin and dynamin, molecules with essential roles in several endocytosis pathways [33], [34], [35]. PV1 on plasma membrane did not colocalize with clathrin light chain at the cell surface of WT cells (Fig. 6A). Unsurprisingly, treatment of cells with PitStop2, a cell-permeant amphiphilic inhibitor of clathrin-mediated uptake (CME), did not have a statistically significant effect on the amount of internalized PV1 at 15 and 60 min time points in WT and Cav1−/− cells (Fig. 6B), while inhibiting the uptake of transferrin that is known to undergo internalization in clathrin coated vesicles (Fig. 6E) [36], [37]. Treatment with inhibitors of dynamin Dyngo4a [36] (Fig. 6C) or Dynasore [38] (Fig. 6D) had no effect on PV1 internalization while inhibiting the internalization of transferrin (Fig. 6E). Similarly, inhibition of dynamin by overexpression of dynamin 2(K44A)-EGFP construct encoding a dominant negative form of dynamin 2 [39] had no effect on PV1 internalization at 15 and 60 min (Fig. 6F), while inhibiting the uptake of transferrin (Fig. 6G). Thus, PV1 is internalized via a clathrin- and dynamin-independent pathway.

## Discussion

PV1 is an essential component of endothelial diaphragms and a molecule critical for postnatal survival of mice. A major unanswered question in the interpretation of the dramatic phenotype of PV1−/− mice is whether the absence of diaphragms is the only function of PV1. Here, we addressed this question by combining *in vivo* and *in vitro* approaches in which we looked at PV1 retention on the plasma membrane in the presence and absence of structures capable of forming diaphragms on the surface of endothelial cells. We found that, in the absence of structures capable of forming diaphragms, PV1 protein is transported to the cell surface followed by rapid internalization and degradation. In our model (Fig. 6H), PV1 is delivered via the secretory pathway to the plasma membrane where PV1 is incorporated into the diaphragms formed on subcellular structures such as caveolae, fenestrae and TEC. PV1 molecules, which are not part of the diaphragms, are rapidly internalized by a clathrin- and dynamin-independent pathway and degraded into lysosomes. In summary, our results strongly suggest that the only role of PV1 is to form diaphragms on the cell surface.

The magnitude of PV1 protein level reduction as a result of caveolae removal was different in the lungs and kidneys. The observation of reduced lung PV1 protein level was in accord to a previous report [21]. The reduced PV1 protein level in the lung microvascular ECs of Cav1−/− mice could not differentiate the roles of Cav1 and/or caveolae in regulation of cellular PV1 amounts. To define whether the reduced PV1 protein level was due to the absence of Cav1 *per se* or to the absence of caveolae, we analyzed another mouse model in which caveolae do not form, namely cavin-1−/− mice. In cavin-1−/− mice Cav1 is still expressed and transported to the plasma membrane but it does not induce caveolae formation and is rapidly internalized and degraded [19], [40]. PV1 protein level in lung ECs of cavin 1−/− was reduced as compared to WT demonstrating that PV1 expression is down regulated by the absence of caveolae. In contrast to the lungs, PV1 protein level in kidneys was only slightly reduced in Cav1−/− compared to WT consistent with the fact that caveolae represent only a minority of the structures capable of forming diaphragms in the kidney microvascular ECs. TEM analysis of the Cav1−/− kidney demonstrated that the numbers of fenestrae and TEC are unchanged from WT, whereas caveolae were absent. Thus, the drop in PV1 protein level in different organs as a result of Cav1 knockout correlated with the level of reduction in the number of structures capable of forming diaphragms.

To gain insight into the mechanism responsible for PV1 decrease as a result of caveolae removal, we have employed an *in vitro* system of cultured lung microvascular ECs isolated from Cav1−/−, Cav1-ECRC and WT mice. As shown before [27], the WT lung microvascular ECs have only caveolae and no fenestrae and TEC. Similar to *in situ*, the lack of caveolae in Cav1KO-MLEC correlates with low total cellular PV1 level. Thus, our data show, both *in situ* and *in vitro*, that the absence of structures capable of forming diaphragms leads to a decrease of PV1 protein level.

The low PV1 protein level in absence of caveolae could be due to decreased production, increased degradation rate or a combination of the two. Our data showed that the decrease in PV1 protein level was not due to diminished PV1 transcription (both *in situ* and in culture) or translation rates, which are similar in WT and Cav1−/− ECs in culture. Moreover the N-glycosylation rate of PV1 is similar in WT and Cav1−/− MLECs, demonstrating similar progression through the secretory pathway components such as the endoplasmic reticulum and the Golgi complex, where the N-glycosylation is completed and from where PV1 is delivered to the cell surface. Our data in the kidney capillary ECs *in situ* and in MLECs show that PV1 does not require caveolae or Cav1 in order to be delivered to the cell surface. Previous surface biotinylation experiments showed that most (>95%) of the cellular PV1 in ECs occurs at the cell surface, all of it in its fully N-glycosylated form [11]. Similarly, in our experiments PV1 is delivered to the cell surface and >95% of PV1 is fully N-glycosylated in both WT and Cav1−/− ECs (and Cav1ECRC). Thus, the production and trafficking of PV1 protein to the cell surface do not seem to be affected by the absence of caveolae and as such are not the cause of the decrease in PV1 protein levels. Still PV1 protein level on plasma membrane of Cav1−/− ECs is significantly lower as compared to WT. This is not due to proteolysis and shedding but rather to an accelerated rate of internalization followed by degradation, as shown by pulse metabolic labeling and experiments using lysosomal degradation inhibitors. Altogether, these data argue that while PV1 arrives at the plasma membrane in normal fashion, it is not retained on the cell surface in the absence of structures capable of forming diaphragms (*i.e.* caveolae) being internalized and targeted toward lysosomal degradation.

Our *in situ* experiments strongly suggest a role for fenestrae and TEC similar to caveolae in retaining PV1 at the plasma membrane. Technical limitations do not allow us to use cell culture methods to confirm the role of fenestrae and TEC in retention of PV1 on the cell surface. Induction of fenestrae and TEC can be achieved by treatments with phorbol esthers [11] and VEGF [12], [41] but these treatments also cause an increase PV1 mRNA transcription. Other methods using actin cytoskeleton disruptors [12] perturb many cellular functions aside from endocytosis. Therefore, we can rely only on our *in situ* data to draw conclusions on the role of fenestrae and TEC in retention of PV1 at the plasma membrane.

The internalization of PV1 occurs via a clathrin- and dynamin-independent pathway in both WT and Cav1−/− lung ECs. These criteria exclude clathrin-mediated uptake and also exclude the dynamin-sensitive clathrin-independent pathways such as caveolae [42], [43], [44], the RhoA controlled IL-2R pathway [45] and the growth factor induced macropinocytosis [46]. Similar dynamin-insensitive non-clathrin endocytic pathways have been already described such as the CLIC pathway [47], the pathway for syndecan 4 internalization in ECs [48], the pathway induced by multivalent toxins and SV40 virus [49], [50] and the pathway for CD36 and oxidized low density lipoprotein particles [51], to name a few. Our data suggest that to enter ECs, PV1 needs to dissociate from caveolae following some form of signal or following to caveolae disassembly. Finally, the faster rate of PV1 internalization in the Cav1−/− ECs suggests that: *i)* Cav1/caveolae have a direct inhibitory effect on the PV1 uptake pathway in WT ECs, or *ii)* PV1 uptake acts in constitutive manner and the rate of dissociation of PV1 from caveolae controls the rate of its internalization. PV1 is internalized via a pathway that feeds into the endolysosomal system leading to its degradation in the lysosomes. Further work will elucidate the details of PV1 internalization and trafficking.

In summary, we found that PV1 is faithfully associated with the diaphragms formed in the neck of caveolae, fenestrae and TEC. In the absence of the structures capable of forming the diaphragms, PV1 undergoes rapid internalization and degradation. These findings will prove to be important in future investigations on the cause of metabolic defects observed in PV1 knockout mice [14] as well as on the significance of PV1 upregulation observed during angiogenesis [41], [52] and inflammation [53], [54] (reviewed in [1], [7]).

## Materials and Methods

### Materials

Cell culture regents were from Lonza (Walkersville, MD). Pharmacological inhibitors bafilomycin A1 (from *Streptomyces griseus)*, Dynasore (3-Hydroxynaphthalene-2-carboxylic acid-(3,4-dihydroxybenzylidene)-hydrazide), E-64D ((2S,3S)-*trans*-Epoxysuccinyl-L-leucylamido-3-methylbutane Ethyl Ester) and leupeptin were from EMD Biosciences/Merck (San Diego, CA), Dyngo 4a, PitStop2 and PitStop2 negative control were from Ascent Scientific/Abcam (Princeton, NJ), whereas epoxomycin and *clasto*-Lactacystin β-Lactone were from Boston Scientific (Natick, MA).

### Mice

Caveolin 1−/− (Cav1−/−) mice on C57Bl/6 background (strain B6.Cg-*Cav1^tm1Mls^*/J) [55] were obtained from The Jackson Laboratory (Bar Harbor, Maine). The mice were maintained by heterozygous (Cav1+/−) cross breeding to generate to generate Cav1−/−, Cav1+/− and WT littermates. Every 5 generations Cav1−/− mice were bred to C57Bl/6J mice from Jackson Laboratory. Cavin-1−/− mice on C57Bl/6 background were from Dr. P. Pilch, Boston University [20].

### Cells

Mouse polyoma virus middle T antigen immortalized mouse lung ECs (MLEC) obtained from Cav1KO (MLEC-Cav1KO), Cav1 ECRC (MLEC-Cav1 ECRC) and WT (MLEC-WT) mice, as described [27], [56], [57], [58]. The cells were flow sorted for surface expression of both CD31 and PV1 using a FACSAria sorter (BD Biosciences), as described below. All mouse lung ECs were cultured on plastic in MLEC growth medium consisting of endothelial growth medium 2 (EGM2) (Lonza, Walkersville, MD) supplemented with 15% heat inactivated fetal bovine serum (Hyclone), 100 µg/ml penicillin, 100 µg/ml streptomycin and 100 µg/ml glutamine (Invitrogen, Carlsbad, CA). HUVEC were obtained from Lonza and were cultured in EGM2 medium. The hybridoma secreting the rat anti-mouse PV1 IgG2a mAb MECA-32 [6], [59] were from the Developmental Studies Hybridoma Bank, University of Iowa.

### Antibodies

Rabbit anti caveolin 1 pAb (cat# 610060) and mouse anti –PTRF (cavin1) mAb, clone #4 (Cat# 611259) were purchased from BD Bioscience (San Diego, CA). Rat anti-mouse CD31 (PECAM-1) clone MEC13.3 – APC (Cat# 102510) was from BioLegend. Mouse anti-beta-actin mAb (AC40) was from Sigma (St.Louis, MO). Rabbit anti ERK1/2 mAb was from Cell Signaling (Beverly, MA). Goat anti-CD31 (PECAM-1) pAb (M-20, cat# sc-1506) and goat anti-Cdh5 (VE Cadherin, CD146) pAb (C-19, cat# sc-6458) were from Santa Cruz. The unlabeled and HRP-conjugated rabbit anti-chicken IgY and the goat anti-mouse IgG-HRP were from Biodesign (Kennebunk, ME). Rat anti-mouse PV1 IgG2a mAb, clone MECA-32 mAb was produced in serum free media by BioXCell, Lebanon, NH. *Chicken anti-mouse PV1C pAb* was raised in chickens against the last 12 aa of mouse PV1 C terminus, as described in the past for chicken anti-human PV1C pAb [8], [11].

### Primary antibody labeling with fluorophores

Affinity purified primary antibodies rat anti-mouse PV-1 mAb clone MECA-32 and chicken anti-mouse PV-1C pAb, were labeled with either Alexa (488, 568 or 647) fluorophores (Molecular Probes, Invitrogen), as per manufacturer's instructions.

### Cell Sorting

MLEC-Cav1KO, MLEC-Cav1 ECRC and MLEC-WT cells were cultured in 10 cm dishes to confluence. The cells were incubated (30 min, 10°C) live with 1∶500 anti-CD31-APC (BioLegend) and anti-PV1-AF488 mAb clone MECA-32 (1 µg/ml) diluted in MLEC growth medium. The excess antibody was rinsed (3×5 ml) with sterile PBS (Invitrogen) and the cells dissociated nonenzymatically with cell dissociation solution (Sigma). The gating parameters were set on CD31+/PV1+ positive cells.

### Electron microscopy

Specimen preparation for electron microscopy was done as before [8], [9]. Cav1−/− mice or WT littermates were perfused (10 min, RT) under anesthesia, with oxygenated DMEM through the left ventricle, followed by fixation by perfusion (10 min at RT) with 2.5% glutaraldehyde and 3% paraformaldehyde in 0.1 M sodium cacodylate buffer (pH 7.3). Specimens were taken from different tissues and trimmed into small blocks. The blocks were immersed into fresh fixative (1 h at RT), washed twice (15 min, RT) in 0.1 M cacodylate, postfixed in Palade's OsO_4_ (1 h on ice), en bloc-stained in Kellemberger's uranyl acetate (overnight at RT), dehydrated in graded ethanol, and embedded in LX112 resin (Ladd Research Industries, Burlington, VT). Thin sections (40 nm) with a Leica Ultracut (UC-6) using an ultrasonic oscillating diamond knife (Diatome, US), stained with lead citrate, and examined and photographed under an electron microscope (JEOL 1010).

### Morphometry

Morphometry was done as before [9], [57] on lungs and kidneys obtained from Cav1−/− and WT mice. The measurements were obtained from capillaries found in 15–20 sections per animal per tissue (n = 3 mice per group, 5 blocks per animal, 3–4 sections per block).

In the case of the lung, the number of caveolae per µm of EC length was determined by counting the number of uncoated plasmalemma invaginations in the 50–100 nm size range [6] at the luminal or abluminal front of the ECs in capillaries (*i.e.* blood vessels with <10 µm diameter) [1]. The total membrane length examined was determined by summing the total length of luminal membrane to the total length of abluminal membrane. The data obtained in all animals per group was expressed as number of caveolae per µm endothelial membrane length. Student's *t* test was used to determine statistical significance between WT and Cav1−/− groups.

In the case of the kidney, we have determined the number of caveolae, TEC and fenestrae per µm EC length in the ECs of the peritubullar capillaries following the methodology previously described by Millici, et al., [15]. By this, caveolae, TEC and fenestrae were counted only in the areas in which EC thickness was less than 400 nm, as these are the areas where TEC and fenestrae occur. In the case of uncertainty as to whether a transendothelial opening was a TEC (i.e. with two diaphragms) or a fenestra (i.e. only one diaphragm) we have labeled this as an unknown (U). The data is expressed as the total number of structures found in 45–50 sections from each genotype divided by the membrane length. Because the fenestrae and TEC involve both fronts of the EC plasma membrane (*i.e.* luminal and abluminal) we have considered the total membrane length as an average of the luminal and abluminal plasma membrane.

### Colocalization of PV1 and Cav1 by Total Internal Reflection Fluorescence Microscopy (TIRFM)

MLEC-wt cells were seeded at 50% confluence on glass bottom dishes (MatTek) and were trasnfected with Cav1-EGFP [48], using Fugene 6 (Roche). Twenty four to forty eight hours post transfection the cells were labeled live with 1.5 µg/ml MECA-32-Alexa 568 mAb for 30 min at 4°C in MLEC growth medium, the cells rinsed (3×, RT) in MLEC growth medium and immediately used for live TIRFM.

TIRFM images were acquired live as before [60], using an Olympus IX71 inverted microscope equipped with a temperature-controlled stage set at 32°C, a 1.45 NA 60× TIRFM lens (Olympus), back-illuminated electron-multiplying charge-coupled device camera (512×512, 16-bit; iXon887; Andor Technologies), and controlled by Andor iQ software (Andor Technology). Excitation was achieved using a 488-nm and a 514-nm line of laser, and exposure times were 0.1–0.2 s and acquired at 0.5–4 Hz. The calculated evanescent field depth was 100 nm.

Due to optical characteristics of the two wavelengths leading to an uneven signal in the two wavelengths in TIRFM, the colocalization was done by scoring puncta positive for the two labels and not by the usual thresholding and calculation of the colocalization index but manually.

### Transfections

MLEC-WT were seeded at 70–90% confluence in 12 well plates and transfected with different DNA constructs using Superfect (Qiagen), as per manufacturer's instructions. The DNA constructs were as reported before [48]: EGFP-clathrin light chain from J. Keen (Thomas Jefferson University, Philadelphia, PA) and dynamin 2 wt-EGFP and dynamin 2(K44A)-EGFP in pEGFP-N1 vector from M. McNiven [39] (Mayo Clinic, Rochester MN). pEGFP-N1 empty vector was from Clontech. Forty-eight hours post transfection the cells were labeled with fluorescent anti-PV1 and processed for either confocal microscopy or flow cytometry.

### Confocal microscopy

MLEC were serum starved (2 h, 37°C, EBM2), labeled (30 min, 10°C, EBM2+1%BSA) with fluorescent anti-PV1 (5 µg/ml) and rinsed (3×, RT) in PBS containing calcium and magnesium (PBS-CM) and chased (37°C, MLEC growth medium) for different amounts of time. After 0, 15, 60 and 120 min the cells were rinsed (2×30 sec, RT) at low pH to facilitate detachment of non-internalized antibodies, rinsed 1× in neutral PBS, fixed (10 min, RT) in 4% paraformaldehyde in PBS-CM, rinsed again in PBS-CM containing DAPI, mounted in PermaFluor (Thermo Fisher) mounting medium and examined by confocal fluorescence microscopy using a Zeiss 510 Meta confocal system equipped with a 63× oil immersion objective and appropriate lasers. Stacks of images were acquired with the pinhole set at 1 Airy unit and processed using ImageJ software. For PV1 internalization, stacks were transformed through the maximum intensity projection function to obtain global images of the cells. Figures were prepared using Adobe Photoshop and Adobe Illustrator CS3 software.

### Isolation of total membranes from lungs and kidneys

Lung and kidney membrane lysates were obtained from WT, Cav1^−/−^, Cav1^+/−^ and cavin-1^−/−^ mice, as described in the past [9]. The mice were anesthetized with a mixture of ketamine : xylazine : acepromazine (3∶1∶0.25). The lungs and the kidneys were immediately flushed free of blood by perfusion (10 min, 25°C) with oxygenated phenol-red free HBSS, via the pulmonary artery or the left ventricle, respectively. The organs were freshly collected, weighed, minced and homogenized (20 strokes, Teflon pestle-glass Thomas type BB homogenizer) in an ice-cold buffer (1∶4/w∶v) containing 25 mM Hepes, pH 7.2, 250 mM sucrose, 2 mM MgCl_2_ and a protease inhibitors cocktail (10 µg/ml each leupeptin, pepstatin, o-phenantrolin, E-64 and 1 mM PMSF). The homogenate was filtered through 53 µm nylon net and centrifuged for 15 min at 500×g to yield a nuclei/cell debris pellet and a postnuclear supernatant (PNS). The PNS was further fractionated by centrifugation (1 h, 4°C, 100,000×g, using a TLA45 rotor) in a total membranes pellet and a cytosolic supernatant. The membrane pellet was solubilized in 200 µl 10 mM Tris, pH 6.8, 0.5%SDS, and protease inhibitors (Sigma). Protein concentration was determined by a bicinchoninic acid method (Pierce, Rockford, IL/USA) using BSA standards standards prepared in solubilization buffer, as described previously [10], [11]. Equal amounts of protein (20 µg) were adjusted to 1× reducing SDS-PAGE sample buffer, boiled for 5 min, resolved by 12% or 8% SDS-PAGE, transferred to PVDF membrane and probed by immunoblotting. Antibodies used were either the rat anti-mouse PV1 MECA-32 mAb, the chicken anti-mouse PV1C pAb described here, rabbit anti caveolin 1 pAb, mouse anti-Cavin-1/PTRF, goat anti CD31 pAb, goat anti-VE Cadherin pAb and rabbit anti-Cavin 2/SDPR (Abcam). The lung membrane lysates were obtained from WT, Cav1KO, Cav1^+/−^ and Cavin-1−/− mice.

### MLEC fractionation

CD31 and PV1 positive MLEC-WT, -Cav1KO or -Cav1-ECRC grown to confluence in 2×15 cm dishes were scrapped and collected by centrifugation in tubes, the pellets resuspended in 1 ml ice-cold buffer (containing 25 mM Hepes, pH 7.2, 250 mM sucrose, 2 mM MgCl_2_ and a broad spectrum protease inhibitors cocktail - Sigma), homogenized (4°C, 20 strokes, Teflon pestle-glass Thomas type A homogenizer) followed by sonication (3×30 sec bursts) using a Branson sonicator. The homogenate was fractionated as described above for the lung and kidney membranes.

### RNA isolation

MLEC-wt and MLEC-Cav1KO cells were grown to confluence in quadruplicate in 15 cm Petri dishes. Freshly harvested lungs and kidneys from WT, Cav1^−/−^ and cavin-1^−/−^ mice were used for total RNA isolation. Total RNA was isolated using Trizol (Invitrogen), as per manufacturer's instructions.

### Real-time quantitative PCR

RNA integrity and quality were determined using Bioanalyzer (Agilent) and NanoDrop (Thermo-Fisher). One microgram of total RNA was reverse transcribed using High Capacity cDNA Reverse Transcription Kit (Applied Biosystems). cDNA amplified from 10 ng RNA were used in triplicate for quantitative real-time PCR using Taqman® Gene Expression Assays (ABI) designed for mouse PV1 (PV1/Plvap), Cav1, cavin-1 and Actin B (ActB) mRNA detection and the TaqMan® Gene Expression Master Mix, as per manufacturer's instructions. The PCR was performed on ABI 7500 Real Time PCR System with SDS software. The comparative C_T_ method (2^−ΔΔCT^) of relative quantitation was used to compare the two genotypes.

### PNGase treatment to remove N-linked glycans

Solubilized membrane (Mem) and cytosolic (Cyt) proteins (100 µg) were treated with PNGase F (New England Biological), as per manufacturer's instructions. Controls were incubated in the same conditions except that PNGase F was omitted. The samples were resolved by 8% SDS-PAGe and the proteins transferred to PVDF membrane and immunoblotted with chicken anti-PV1C pAb.

### ^35^S metabolic labeling of MLEC

MLEC-wt and MLEC-Cav1KO cells were grown to confluence in 60 mm dishes. Cells were Met and Cys starved by incubation (2 h, 37°C) in ^35^S labeling medium consisting of EBM medium lacking these Met and Cys (Lonza). 100 µCi of ^35^S Translabel (Perkin-Elmer) consisting of a mix of ^35^S labeled Met and Cys were added to the 35S labeling medium and the cells were incubated for 10 min at 37°C to allow for the ^35^S-labeled aminoacids to be incorporated in proteins during translation. Mouse PV1 has 8 methionines and 10 cysteines in its primary sequence. After 2 washes in EBM2, the cells were chased for 0, 5 min, 15 min, 30 min or 1, 2, 4, 8, 12 and 24 h when the cells were rinsed in PBS, collected by scrapping in 1 ml solubilization buffer [1% Triton X-100 in 10 mM Tris-Cl, pH 7.4, 150 mM NaCl and protease inhibitors cocktail (Sigma cat# P8340)] followed by incubation (4°C, 2 h) with end over end rotation to complete the solubilization, These conditions are known to efficiently solubilize PV1 [8]. The samples were centrifuged (1 h, 4°C, 100,000×g) to remove the insoluble material as a pellet. The supernatant, containing the solubilized PV1, was added to 50 µl (settled gel) of chicken anti-mouse PV1 directly coupled to AffiGel 10 beads, as described before [8]. Beads coupled with preimmune chicken IgG were used as controls. The samples were incubated (o/n, 4°C) by rotation after which the beads were collected by centrifugation (5 min, 4°C, 300×g) and washed 3 times at 4°C with solubilization buffer. PV1 bound to the beads was solubilized in SDS-PAGE sample buffer, resolved by 8% SDS-PAGE, the gel treated with Amplify (GE Healthcare), vacuum dried and exposed to a multipurpose standard (MS) phosphor storage screen (Kodak). The signal was imaged using a Typhoon 9400 scanner (Molecular Dynamics, GE Healthcare) and quantified using ImageJ or GelEval v1.35 (FrogDance, UK) software. Data from three separate experiments were used to obtain the degradation curves.

### Flow Cytometry

Labeled cells were analyzed by either using a FacsCalibur or a CANTO flow cytometer controlled by either CellQuest or DIVA software, respectively (BD Biosciences). The data analysis was carried out using FlowJo (Tree Star, Ashland, OR) software. Each experiment had 4–8 samples per time point and was repeated at least three times. Median fluorescence from at least 10,000 live cells was calculated in each sample. Statistical significance was calculated using Student's *t* test.

### Evaluation of cell surface PV1 levels by flow cytometry

Either MLEC-wt or MLEC-cav1KO cells were labeled live and while adherent with 1.5 µg/ml MECA-32-Alexa 647 mAb for 30 min at 4°C in MLEC growth medium. The cells were rinsed (3×, RT) in PBS and non-enzymatically detached using EDTA (Cell Dissociation Solution, Sigma). The cells were mixed with an equal volume of 1% BSA in PBS, and kept on ice in the dark until examined by flow cytometry.

### Evaluation of PV1 internalization rate by flow cytometry

Prior to the experiment the MLEC-wt and MLEC-Cav1KO cells were serum-starved (2 h, 37°C) in serum-free endothelial basal medium 2 (EBM2) (Lonza), followed by labeling (30 min, 10°C) with fluorophore coupled rat anti-mouse PV1 MECA-32 mAb (1.5 µg/ml) in EBM2 supplemented with 2% BSA. After washing (3×, EBM2) the excess primary antibody off, the cells were incubated with full MLEC growth medium at 37°C for the indicated periods of time to allow for the internalization of the antibody. To determine the internalized fraction, the cells were washed once (30 s, RT) in acidic PBS, pH 2.5, once in neutral PBS and detached by incubation (10 min, 37°C) in a mixture of trypsin/EDTA (Lonza). The combination of acid wash and the trypsin treatment were very effective in removing the surface anti-PV1, demonstrated on separate control samples incubated at 4°C.

To determine the initial surface pool of PV1, cells were incubated with fluorescent anti-PV1 as above, rinsed in neutral PBS and non-enzymatically detached using EDTA (Cell Dissociation Solution, Sigma). These conditions do not disrupt the anti-PV1 - PV1 interaction on cell surface.

The cell suspensions were mixed with an equal volume of 1% BSA in PBS, and kept on ice in the dark until examined by flow cytometry. The average median fluorescence was calculated from each time point and the percentage of internalized PV1 was calculated from the ratio of internalized/initial anti-PV1 signal. Fluorescent rat IgG2a was used as isotype control for MECA-32 antibody.

### Evaluation of PV1 degradation pathway

Equal numbers of MLEC-WT and MLEC-Cav1KO were seeded into 6 cm dishes at 90% confluence the evening before and cultured in full growth medium until the next day when the medium was replaced with MLEC growth medium containing either proteasome inhibitors (*i.e.* epoxomycin or clasto-Lactacystin beta Lactone), lysosome inhibitors (*i.e.* leupeptin, E64-D or bafilomycin A1) or DMSO vehicle. The cells were further incubated for 4 h, 8 h or 24 h at 37°C in a cell culture incubator with 5%CO2 atmosphere. The final inhibitor concentrations obtained from 1000-fold concentrated stocks in DMSO were as follows: 2 µM epoxomycin, 10 µM *clasto*-Lactacystin β-Lactone, 10 µM E-64D, 50 µM leupeptin, 1 µM or 10 µM bafilomycin A1. At the end of the experiment cells were rinsed (2×, RT) in 5 ml PBS and then solubilized (1 h, 4°C) in .5 ml RIPA buffer (1% Triton X-100, 0.4% sodium deoxycholate, 0.1% sodium dodecyl sulfate, 150 mM NaCl in 25 mM Tris, pH7.6) with protease inhibitors. The samples were centrifuged (1 h, 4°C, 100,000×g) to remove the insoluble material as a pellet. The supernatant was transferred to a fresh tube and used to determine the protein concentration using the BCA assay and standards made in RIPA buffer. Equal amounts (20 µg) of proteins from different samples were resolved by 8% SDS-PAGE, transferred to PVDF membranes and immunoblotted with either rat-anti-mouse PV1 mAb MECA-32 or chicken anti-mouse PV1C pAb, as described [10], [11].

### Evaluation of the effect of dynamin and clathrin inhibitors on PV1 internalization

To determine the effects of clathrin mediated uptake inhibitor PitStop2 [61] or the dynamin inhibitors Dynasore (10 and 80 µM) [38] or Dyngo4a (30 µM) [36] on PV1 internalization, cells were serum-starved (2 h, 37°C) in serum-free endothelial basal medium 2 (EBM2) (Lonza), followed by labeling (30 min, 10°C) with Alexa647-MECA-32 mAb (1.5 µg/ml) in EBM2 supplemented with either: 25 µM PitStop2 (Ascent Scientific), 25 µM PitStop2 negative control compound (Ascent Scientific), 10 µM or 80 µM dynasore (EMD Chemicals) or 30 µM Dyngo4a (Ascent Scientific). Negative controls consisted of cells not treated or treated with DMSO vehicle (1 µl/ml medium). After washing (3×, EBM2) the excess primary antibody off, the cells were incubated (37°C, 15 min or 60 min) with either EMB2 or full MLEC growth medium supplemented with inhibitors or vehicle. For each inhibitor we have determined the total surface PV1 signal at t0 min, t15 min and t60 min by nonenzymatic digestion as well as the internalized fraction using the combination of acid wash and trypsin/EDTA, as described above. For each inhibitor the samples were run in quadruplicate in three separate experiments. Because protein in the medium might inactivate the inhibitors, for each inhibitor the assay was carried out in absence and presence of protein in the medium (EBM2 vs. full growth MLEC medium).

Positive controls for each inhibitor effectiveness consisted of Alexa647 labeled human transferrin or EGF (Invitrogen, Molecular Probes). Serum starved (o/n, 37°C, EBM2+1%BSA) endothelial cells were incubated (30 min, 10°C) in serum-free medium supplemented with inhibitors and either 25 µg/ml transferrin-F647 or 1 µg/ml biotinEGF-streptavidin-Alexa647, the excess label washed away, the cells incubated (10 min, 37°C) in growth medium with inhibitors to allow internalization of the surface bound label, when the cells were acid washed, resuspended using trypsin/EDTA and examined by flow cytometry.
